# Epigenetic Contribution and Genomic Imprinting Dlk1-Dio3 miRNAs in Systemic Lupus Erythematosus

**DOI:** 10.3390/genes12050680

**Published:** 2021-05-01

**Authors:** Rujuan Dai, Zhuang Wang, S. Ansar Ahmed

**Affiliations:** Department of Biomedical Sciences and Pathobiology, Virginia-Maryland College of Veterinary Medicine (VMCVM), Virginia Tech, Blacksburg, VA 24061, USA; wzhuang@vt.edu

**Keywords:** epigenetics, DNA methylation, microRNA, genomic imprinting, Dlk1-Dio3, systemic lupus erythematosus

## Abstract

Systemic lupus erythematosus (SLE) is a multifactorial autoimmune disease that afflicts multiple organs, especially kidneys and joints. In addition to genetic predisposition, it is now evident that DNA methylation and microRNAs (miRNAs), the two major epigenetic modifications, are critically involved in the pathogenesis of SLE. DNA methylation regulates promoter accessibility and gene expression at the transcriptional level by adding a methyl group to 5′ cytosine within a CpG dinucleotide. Extensive evidence now supports the importance of DNA hypomethylation in SLE etiology. miRNAs are small, non-protein coding RNAs that play a critical role in the regulation of genome expression. Various studies have identified the signature lupus-related miRNAs and their functional contribution to lupus incidence and progression. In this review, the mutual interaction between DNA methylation and miRNAs regulation in SLE is discussed. Some lupus-associated miRNAs regulate DNA methylation status by targeting the DNA methylation enzymes or methylation pathway-related proteins. On the other hand, DNA hyper- and hypo-methylation are linked with dysregulated miRNAs expression in lupus. Further, we specifically discuss the genetic imprinting Dlk1-Dio3 miRNAs that are subjected to DNA methylation regulation and are dysregulated in several autoimmune diseases, including SLE.

## 1. Introduction

Systemic lupus erythematosus (SLE) is a chronic, debilitating, systemic autoimmune disease. It is clear that genetic factors play a critical role in the predisposition of SLE. Genome-wide association studies have identified many genetic risk factors associated with human and murine lupus [1,2,3]. These genetic risk factors include gene defects in complement components such as C1q, C2, and C4, genes that regulate toll-like receptor (TLR)s/type I interferon (IFN) signaling pathways such as *TLR7/8*, three prime repair exonuclease 1 (*TREX1*), interferon regulatory factor 5 (*IRF5*), signal transducer and activator of transcription 4 (*STAT4*), and the genes that regulate T and B cell signaling pathways such as protein tyrosine phosphatase, non-receptor type 22 (*PTPN22*), programmed cell death 1 (*PDCD1*), B cell scaffold protein with ankyrin repeats 1 (*BANK1*), and B lymphocyte kinase (*BLK*) [1,2,3,4]. Nevertheless, the sole genetic contribution cannot address the complexity of disease etiology and manifestation, especially the discordance of lupus incidence in genetically identical twins [5,6]. Genetic, epigenetic, hormonal, and environmental factors are multiple factors that interact in a complex manner in the susceptibility and the onset of autoimmune diseases. The incidence of many autoimmune diseases such as SLE, Sjögren’s syndrome, and autoimmune thyroiditis show strong female prevalence [7]. While the precise reasons for the female predominance of autoimmune diseases are not fully clear, it could be due to the intrinsic differences in sex chromosomes (XX versus XY), sex hormones (estrogens versus testosterone), and/or differential responses to extrinsic cues such as environmental exposures [8,9]. Moreover, epigenetic factors such as DNA methylation and non-coding small RNAs (microRNAs) have been increasingly recognized in recent studies as key contributors to SLE [10,11] and other autoimmune diseases [12,13,14].

DNA methylation is a biochemical process that adds a methyl group to 5′ cytosine within a CpG dinucleotide to control DNA accessibility and gene expression. It is generally accepted that hypermethylated DNA sequences at the gene regulatory regions suppress gene expression at the transcriptional level, while hypomethylated DNA sequences permit the activation of gene expression. DNA methylation is involved in the regulation of many critical biological processes including embryonic development, X-chromosome inactivation (XCI), and genomic imprinting [15]. Aberrant methylation at the whole genome level or the specific genes has been identified in many human diseases [16], including but not limited to different cancer types such as breast cancer [17,18], lung cancer [19], and leukemia [20]; metabolic disorders such as type 2 diabetes mellitus [21,22]; neurological disorders such as autism spectrum disorder [23] and Parkinson’s disease [24]; and autoimmune disorders such as rheumatoid arthritis (RA) [25], multiple sclerosis (MS) [26], and SLE [27,28,29].

miRNAs are small, non-protein coding RNAs that suppress gene expression at the post-transcriptional level mainly via translation inhibition or inducing messenger RNAs (mRNAs) degradation [30,31]. While not common, miRNA-mediated translation activation and transcriptional regulation of gene expression have also been observed [32,33]. In the last three decades, miRNAs have emerged as a key epigenetic regulator in various human diseases [34,35,36], including SLE [11,37]. In this review, we present the current understanding of the epigenetic regulation in SLE, emphasizing the interplay of DNA methylation and miRNA regulation. Further, we specifically discuss the dysregulation of genetic imprinting Dlk1-Dio3 miRNAs in autoimmune disease, which may provide us with a new perspective for understanding the epigenetic mechanism and the role of genomic imprinting in autoimmune disease.

## 2. DNA Methylation and SLE

A series of elegant studies from Dr. Richardson’s group established the significant contribution of CD4^+^ T cell DNA hypomethylation to lupus [38,39]. They have reported that inhibition of DNA methylation in vitro induced autoreactivity in cloned CD4^+^ T cells [40,41], and that T cells from SLE and rheumatoid arthritis (RA) patients have reduced DNA methylation levels when compared to that of T cells from healthy controls [42]. They further demonstrated that adoptively transferring demethylated normal human and murine CD4^+^ T cells was capable of inducing lupus-like disease in syngeneic recipient mice [43]. This study strongly suggests the vital contribution of global DNA hypomethylation in CD4^+^ T cells to lupus. The studies from this group also identified numerous lupus-related genes including integrin subunit α L (*ITGAL*, or *CD11a*) [44], CD40 ligand (*CD40LG*) [45], tumor necrosis factor ligand superfamily 7 (*TNFSF7* or *CD70*) [46], and perforin (*PRF1*) [47], which are hypomethylated in human lupus T cells, correlating with the overexpression of these genes in SLE patients.

With the advancement of technology, researchers can profile the DNA methylation status at the genome-wide level to determine the correlation between DNA methylation and gene expression changes in lupus cells [5,29,48,49,50,51,52]. Javierre et al. profiled the DNA methylation of 807 CpG-containing gene promoters in DNA samples from whole blood cells of monozygotic twins discordant for SLE, RA, and dermatomyositis [5]. They found that only monozygotic twins for SLE had widespread changes in the DNA methylation pattern of 49 genes, of which many are associated with immune functions. These changes were in parallel with the global DNA hypomethylation in the SLE twins. By using the Illumina Infinium HumanMethylation 27 array, two different research groups profiled the methylation of 27,578 CG sites located within the promoter regions of 14,475 genes in peripheral blood CD4^+^ T cells from different groups of SLE patients and healthy controls [29,48]. Jeffries et al. identified 232 hypomethylated genes and 104 hypermethylated genes in lupus CD4^+^ T cells [29]. Lin et al. reported that 2165 genes had significant methylation changes between the SLE and control group [48]. Ingenuity pathway analysis of these differentially methylated genes revealed a significant enrichment of the network of cellular movement, hematological system development and function, and immune cell trafficking [48]. Lin et al. further confirmed the hypomethylation of IL-10 and IL-1R2 in CD4^+^ T cells from lupus patients. There was a trend of having IL-10 and IL-1R2 hypomethylation in SLE patients with greater disease severity [48]. In the more recent studies, the Illumina Methylation450 Beadschip array, which measures DNA methylation status over 485,000 CpGs, covering 99% of the RefSeq genes of the human genome, was used to profile the DNA methylation in different immune cell subtypes from SLE patients [49,50,51,52]. Significantly, these studies commonly revealed the hypomethylation of signature IFN-related genes in different immune cell subsets from lupus patients. Coit et al. performed a genome-wide DNA methylation analysis of naïve CD4^+^ T cells from two independent groups of lupus patients and matched healthy controls [49]. Of the 47 differentially methylated genes identified in lupus naïve CD4^+^ T cells, 35 genes (75%) were hypomethylated. The majority of hypomethylated genes (21 out of 35) were IFN-regulated, which include interferon induced protein with tetratricopeptide repeats 1 (*IFIT1*), *IFIT3*, MX dynamin like GTPase 1 (*MX1*), *STAT1*, interferon induced protein 44 like (*IFI44L*), ubiquitin specific peptidase 18 (*USP18*), tripartite motif containing 22 (*TRIM22*), and bone marrow stromal cell antigen 2 (*BST2*). The hypomethylation of IFN-related genes such as *IFI44L* and *BST2* was correlated with the overexpression of these two genes in total CD4^+^ T cells from lupus patients. Nevertheless, the hypomethylation of IFN-related genes in lupus naïve CD4^+^ T cells did not induce the overexpression of these genes in lupus naïve CD4^+^ T cells, and it was also not correlated with the disease activity [49]. These data reveal an epigenetic “poising” of interferon-regulated genes in lupus naïve CD4^+^ T cells, suggesting a mechanism for type-I interferon hyper-responsiveness in lupus T cells [49]. Almost at the same time, by profiling the genome-wide methylation status of CD4^+^ T, CD19^+^ B, and CD14^+^ monocytes from 49 SLE patients and 58 healthy controls, Absher et al. found a common hypomethylation feature near the genes involved in Type I interferon signaling in lupus CD4^+^ T, CD19^+^ B, and CD14^+^ monocytes [50]. The hypomethylation of IFN-related genes is consistent with the previous finding of the upregulation of IFN-regulated genes in SLE patients [53], especially during flares of the disease. The hypomethylation of IFN genes in SLE patients at both active and quiescent stages suggested that the DNA methylation-mediated hypersensitivity to IFN in lupus is independent of the disease activity and circulating IFN levels. The overall DNA hypomethylation and robust DNA demethylation of IFN signature genes such as *MX1*, *IFI44L*, interferon induced transmembrane protein 1 (*IFITM1*), Poly(ADP-ribose) polymerase family member 9 (*PARP9*), *IFIT3*, DExD/H-Box helicase 60 (*DDX60*), lymphocyte antigen 6 family member E (*LY6E*), and interferon-stimulated gene 15 (*ISG15*) has also been identified in lupus neutrophils [51]. In addition, the hypomethylation TLR signaling pathway genes such as eukaryotic translation initiation factor 2 α kinase 2 (*EIF2AK2*) and interleukin 1 receptor associated kinase 3 (*IRAK3*) in lupus peripheral blood mononuclear cells (PBMCs), which correlated with the enrichment of differentially expressed genes involved in IFN and TLR signaling pathways, has also been reported [52]. Together, these studies demonstrate an important contribution of DNA hypomethylation to the altered gene expression and immune function in lupus.

## 3. Mechanism of DNA Hypomethylation in SLE

**Figure 1:**
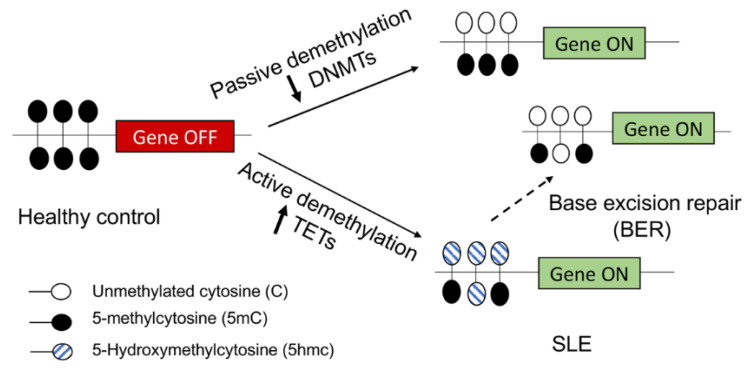
DNA demethylation mechanism in SLE. In healthy controls, the specific lupus-related gene is turned off by the methylation of cytosine (5-mC) in the CpG dinucleotide in the gene promoter. In SLE, the lupus-related gene is turned on by the demethylation of the gene promoter. In the passive DNA demethylation pathway, due to reduced DNMTs expression and/or activity, the 5-mC in the gene promotor is passively diluted following DNA replication. In the active DNA demethylation pathway, 5-mC is actively oxidized into 5-Hydroxymethylcytosine (5hmc), which can be further oxidized into 5-formylcytosine and 5-carboxylcytosine by TETs, and then repaired into unmethylated cytosine (C) through the base excision repair (BER) process.

The important contribution of DNA hypomethylation to lupus is now much appreciated. However, there is a dispute about the mechanisms underlying DNA hypomethylation in lupus cells [27,54]. In mammalian cells, DNA methylation is dynamically established, maintained, and removed by DNA methylation enzymes, DNA methyltransferases (DNMTs), and demethylation enzymes *ten-eleven translocation* proteins (TETs) [55,56]. DNA demethylation can occur through either a passive demethylation pathway due to reduced expression/activity of DNMTs or active demethylation mediated by TETs (Figure 1).

Among the three major conserved DNMTs, DNMT1 is mainly responsible for maintaining DNA methylation during DNA replication, and DNMT3a/3b is mainly responsible for de novo DNA methylation. DNA hypomethylation in lupus has been thought to be contributed by the reduced expression/activity of DNMTs (Figure 1). It has been reported that *DNMT1* and/or *DNMT3a/3b* expressions were significantly decreased in human lupus PBMCs [57] and CD4^+^ T cells [58] when compared to those of healthy controls, which correlated with the global DNA hypomethylation in the cells. Nevertheless, other studies demonstrated unchanged *DNMT1* and even increased *DNMT3a/3b* expression in human lupus PBMCs [59] or CD4^+^ T cells [27,60], despite the consistency of DNA hypomethylation in these lupus cells. DNMTs-mediated DNA methylation changes of a specific gene could be cell and tissue specific and signal pathway dependent [54]. In addition to reduced DNMTs-mediated passive demethylation, the TETs-mediated active demethylation pathway has been recently implicated in the regulation of the DNA methylation status in lupus CD4^+^ T cells [54,61,62] (Figure 1). TETs can catalyze 5-methylcytosine (5 mc) to 5-Hydroxymethylcytosine (5hmc), which can be further oxidized and repaired to unmethylated cytosine (C), leading to active DNA demethylation [55,63,64]. A recent study revealed that TET2 and TET3 are significantly increased in human lupus CD4^+^ T cells, correlating with increased 5hmc levels at promoter regions and gene activation in human lupus CD4^+^ T cells [62].

## 4. MiRNA and SLE

The dysregulated miRNAs have been identified in both human and murine lupus, and their critical contributions to disease pathogenesis have been extensively reviewed by us [11,65,66] and others [37,67,68,69]. The signature dysregulated miRNAs in lupus have been implicated in disease pathogenesis by affecting both innate immune and adaptive immune response, leading to abnormal production of inflammatory cytokines/chemokines and hyperactivity of T and B cells [11]. The miR-146a, a key miRNA that plays an important role in regulating both innate and adaptive immune responses, has been reported to be significantly decreased in the PBMCs of SLE patients compared to that of healthy controls [70,71]. miR-146a expression is negatively correlated with disease activity and IFN expression scores in SLE patients. The authors further demonstrated that miR-146a negatively regulates the type I IFN signaling pathway via targeting IRF5 and STAT-1. Decreased miR-146a expression contributed to the enhanced type I IFN signaling in human lupus patients [70]. Others have reported that reduced miR-125a expression in lupus T cells has been implicated in the enhanced production of inflammatory cytokine RANTES in lupus [72]. The downregulation of miR-31 contributed to impaired IL-2 production and signaling in lupus T cells [73], and the upregulation of miR-21 promoted T cell responses in lupus via targeting PDCD4 [74].

Some miRNAs have been reported to be dysregulated in lupus B cells, contributing to lupus pathogenesis by promoting B cell hyperactivity. For example, miR-155, a key player for B cell development and function, was increased in human lupus whole peripheral blood [75], PBMCs [74,76], and CD19^+^ B cells [74], and also in the CD4^+^ T and CD19^+^ B cells from murine lupus-prone MRL-*lpr* mice [77]. The upregulation of miR-155 contributed to B cell-hyperactivity and autoantibody production in lupus by targeting Src homology 2 (SH2) domain containing inositol polyphosphate 5-phosphatase 1 (SHIP1) and sphingosine-1-phosphate receptor 1 (S1PR1) [78,79]. Compared to wild-type MRL-*lpr* mice, the MRL-*lpr* mice with miR-155 deficiency had mild lupus-related autoimmune syndrome with decreased anti-dsDNA and inflammatory cytokine expression production and reduced immune complex deposition and lymphocytes infiltration in the kidney [78,79]. In addition, miR-30a [80], miR-1246 [81], miR-29a [82], and miR-7, miR-21, and miR-22 [83] have also been reported to be dysregulated in human lupus B cells, which contribute to abnormal B cell activity in lupus via the targeting of different key signaling molecules involved in B cell development and activation such as tyrosine-protein kinase Lyn, early B-cell factor 1 (EBF1), Crk-like protein (CRKL), or phosphatase and tensin homolog (PTEN). Furthermore, some miRNAs are implicated in lupus by targeting the DNA methylation pathway [11,13].

## 5. The Interplay of DNA Methylation and miRNA Regulation in SLE

It is evident that DNA methylation and miRNA are mutually regulated by each other, leading to abnormal gene expression in various human disease conditions [13,84]. The reciprocal regulation between DNA methylation and miRNA dysregulation has been documented in different studies [84,85,86,87]. For example, there is a negative regulatory circuit of miR-148a/miR-152 and DNMT1 in human breast cancer cells. The overexpression of DNMT1, a target of miR-148a/miR-152, was responsible for the hypermethylation of the miR-148a/miR-152 promoter and reduced expression of these two miRNAs in breast cancer cells [87].

**Figure 2:**
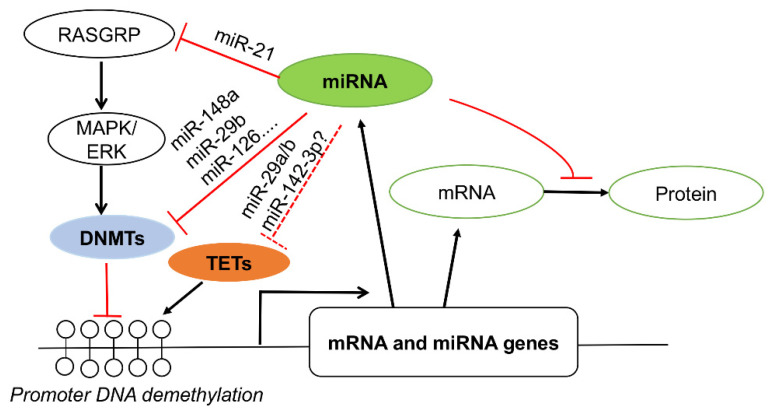
Interplay of miRNAs and DNA methylation in gene expression in SLE. Several dysregulated miRNAs in lupus such as miR-21, miR-148a, miR-29b, and miR-126 can target DNMTs directly and indirectly [88,89,90], leading to gene promoter hypomethylation and activation of mRNA and miRNA genes expression. On the other hand, increased miRNA expression may suppress target mRNA translation to protein. However, whether the dysregulated miRNAs in SLE such as miR-29a/b [82,90] and miR-142-3p [91] target TETs to regulate gene methylation remains unknown (dashed line).

In SLE, several miRNAs including miR-21, miR-148a, miR-126, and miR-29b have been reported to contribute to lupus pathogenesis by targeting the DNA methylation pathway (Figure 2). miR-148a and miR-126 were upregulated in lupus T cells and targeted DNMT1 directly [88,89], whereas upregulated miR-21 and miR-29b targeted DNMT1 indirectly [88,90]. The upregulated miR-21 reduced DNMT1 expression indirectly via targeting RAS guanyl nucleotide-releasing protein 1 (RASGRP1), a mediator of a RAS-MAPK signaling pathway that controls DNMT1 expression and activity [88]. Increased miR-29b in lupus T cells suppressed SP1, a positive regulator of DNMT1, leading to DNA hypomethylation [90]. Overall, the enhanced expression of miR-21, miR-148a, miR-126, and miR-29b in CD4^+^ T cells reduced DNMT1, leading to DNA hypomethylation; overexpression of autoimmune-associated methylation-sensitive genes such as *CD11a*, *CD70*, and lymphocyte function-associated antigen 1 (*LFA-1*); and T/B cell hyperactivity [88,89,90].

On the other hand, the dysregulated miRNAs expression in SLE may be due to the DNA methylation changes in lupus cells. It has been reported that the upregulation of miR-126 in lupus T cells was inversely correlated with the DNMT1 protein expression level [89]. The reduction of miR-142-3p/5p in lupus was correlated with increased histone protein methylation and DNA hypermethylation at the putative regulatory region of the miR-142 precursor [91]. The negative feedback loop between miR-148a and DNA methylation has been reported in human breast cancer cells [87]. Whether there is a feedback loop between upregulated miR-148a and DNA hypomethylation in lupus T cells is not known yet. In addition, some miRNAs such as miR-29a/b/c [92,93] and miR-26a [94] have been reported to target TETs to regulate DNA demethylation. In type I diabetes (T1D), the highly upregulated miR-142-3p in CD4^+^ T cells from human patients and a murine model of T1D interfered with the induction of regulatory T (Treg) cells by targeting TET2, which enhanced the methylation at the conserved non-coding DNA sequence element 2 (CNS2) of the *Foxp3* gene [95]. Inhibition of miR-142-3p enhanced Tregs induction and stability with reduced *Foxp3* CNS2 methylation, leading to reduced islet autoimmunity in non-obese diabetic mice [95]. The interplay between miRNAs and TETs in SLE is expected to be studied soon (Figure 2).

To understand the interplay of different epigenetic mechanisms in lupus gene expression, DNA methylation, mRNA, and miRNA expression profiling analysis at the genome-wide level in CD4^+^ T cells from lupus patients with different clinical manifestations (skin lesion only, skin lesion and renal pathology, or skin lesion, renal pathology, and joint pain) were performed and compared to those of controls [96]. By integration of the DNA methylation and miRNA expression profiling data, the authors revealed a set of miRNAs that are subjected to DNA methylation regulation. Thirty-six upregulated miRNAs in human lupus CD4^+^ T cells were located near the hypomethylated sites, and eight downregulated miRNAs were hypermethylated in the lupus CD4^+^ T cells [96]. In addition, the hypomethylation and overexpression of X chromosome-linked genes such as *CD40LG* and miRNAs have been implicated in the female dominance of lupus [66,97]. Eighteen X-chromosome linked miRNA were highly upregulated in the T cells of female patients with lupus when compared to that of male patients with lupus [97]. Of the 18 miRNAs, 5 miRNAs including miR-98, miR-188, miR-421, let-7f, and miR-503 were induced by the DNA demethylation treatment of the T cells from healthy women, but not men. These data suggest that the upregulation of X-linked miRNAs in women with lupus is likely due to the demethylation of the X chromosome [97]. We have reported that DNA demethylation treatment significantly induced the expression of genomic imprinting Dlk1-Dio3 miRNAs in splenic cells of MRL mice [98]. The substantial upregulation of genomic imprinting Dlk1-Dio3 miRNAs in MRL-*lpr* mice cells was correlated with the global DNA hypomethylation in these cells when compared to that of cells from control MRL mice [98]. Together, these data suggest a critical role of DNA methylation in the regulation of miRNAs expression in lupus.

## 6. Genomic Imprinting Dlk1-Dio3 Locus

**Figure 3:**
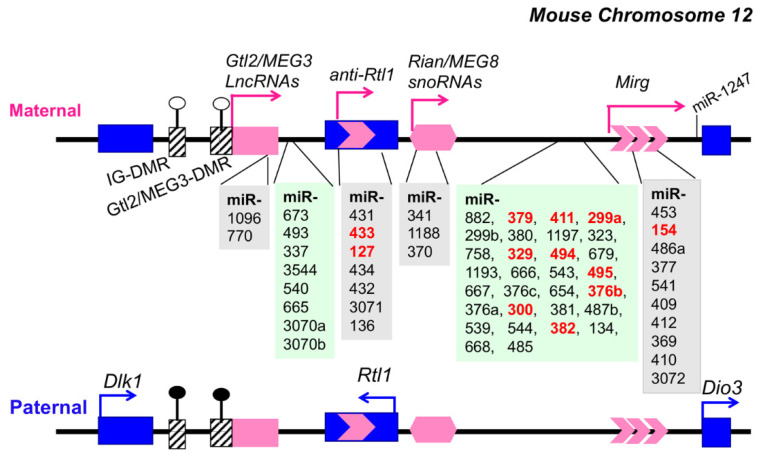
Schematic illustration of genomic imprinting Dlk1-Dio3 miRNAs on mouse chromosome 12. The maternally expressed non-coding genes are shown in pink (top panel), and the paternally expressed protein-coding genes are shown in blue (lower panel). The transcription direction is marked by the arrow. The IG-DMR and Gtl2/MEG3-DMR regions are indicated as rectangles with slashes. The open circle indicates hypomethylation of DMRs and black filled circle means hypermethylated DMRs. The mouse Dlk1-Dio3 harbors sixty-one miRNAs, of which sixty are at the forward strand. One miRNA, miR-1247, is located at the reverse strand. The miRNAs shaded with light gray reside within the *Glt2*, *anti-RTL*, *Rian*, and *Mirg* coding regions. The other miRNAs (shaded in light green) are located at the interval of the aforementioned coding regions. The miRNAs that were identified to be dysregulated in MRL-*lpr* lupus mice [77,98] are bolded in red. The scheme is not drawn to scale.

Genomic imprinting is an epigenetic process that causes allele-specific gene expression of genes based on their parental origin. Some imprinting genes are expressed from the maternally inherited chromosome, while the others are expressed from the paternally inherited copy. The mammalian genomic imprinting Dlk1-Dio3 region is highly conserved and spans over 800 kb on mouse chromosome 12F1 and human chromosome 14q32 [99,100,101,102]. This genomic imprinting region is critically involved in stem cell function, embryonic and placental development, tissue growth, and differentiation [99,100]. The Dlk1-Dio3 locus contains paternally expressed protein-coding genes Delta-like homolog 1 (*Dlk1*), Retrotransposon-like gene 1 (*Rtl1/Mart1*), and the type 3 deiodinase (*Dio3*) (marked in blue) and also maternally expressed non-protein coding genes *Gtl2* (*MEG3* in human), *Rian* (*MEG8* in human), *anti-Rtl1*, and *Mirg* (marked in pink) (Figure 3) [99,100,101,102,103]. Of the three DMRs identified in murine Dlk1-Dio3, the germline-derived intergenic DMR (IG-DMR) plays a primary role in imprinting regulation and functions as an imprinting control region (ICR) [100,104,105,106,107]. The secondary DMR (acquired after fertilization), Gtl2-DMR (or MEG3-DMR in human), located upstream of the Gtl2 promoter and extended into the first intron, also plays a critical role in the regulation of imprinted gene expression [108,109]. The IG-DMR and Gtl2-DMR are hypermethylated at the paternal allele and hypomethylated at the maternal allele (Figure 3). So far, there are 54 miRNAs that have been identified in the human Dlk1-Dio3 region [99], and 61 miRNAs that have been identified in the murine Dlk1-Dio3 locus [101,102], representing the largest miRNA gene cluster in the human and mouse genome. The epigenetic dysregulation of the Dlk1-Dio3 miRNA cluster has been documented in various cancer types, such as melanoma [110], leukemia [111], ovarian cancer [112,113], breast cancer [114], bladder cancer [115], metastatic hepatoblastoma [116], and implicated in tumorigenesis. Recent studies revealed that Dlk1-Dio3 miRNAs are dysregulated in autoimmune disease [98,103] and that the Dlk1-Dio3 locus may play a role in the parent-of-origin effect of autoimmune disease [117].

## 7. Epigenetic Upregulation of Dlk1-Dio3 miRNAs in Murine Lupus

While it remains largely elusive, the parent-of-origin effect has been observed in autoimmune diseases such as multiple sclerosis (MS) [117,118], type I diabetes [119], juvenile idiopathic arthritis (JIA) [120], and rheumatoid arthritis (RA) [119]. The genomic imprinting *Dlk1* gene was identified as a novel risk gene for experimental autoimmune encephalomyelitis (EAE, a rodent model for human MS) [118]. In addition, there is a sex-specific upregulation of the miRNAs from genomic imprinting of the Dlk1-Dio3 locus in human MS patients. The increase of Dlk1-Dio3 miRNAs was observed in the PBMCs from male but not female patients with relapsing-remitting multiple sclerosis (RRMs) [103].

The dysregulation of Dlk1-Dio3 miRNAs has also been identified in both human and murine lupus studies (Table 1). In our previous miRNA microarray profiling study, we found that 11 out of 17 upregulated miRNAs in splenocytes from MRL-*lpr* lupus mice are located at the genomic imprinting Dlk1-Dio3 locus [77]. Similar data were observed in B6-*lpr* mice [77]. Further, by using the Taqman miRNA assay the upregulation of Dlk1-Dio3 miRNAs in whole splenocytes, purified splenic CD4^+^ T and CD19^+^ B cells [77,98], and PBMCs [121] of MRL-*lpr* mice was validated. In addition to the *lpr* lupus model, the upregulation of selected Dlk1-Dio3 miRNAs has also been observed in the splenocytes of the other murine lupus murine models, NZBW_F1_ mice [77] and C3.MRL-Fas^lpr^/J mice [122] (miR-127 and miR-379). The miRNA profiling studies have also identified the upregulation of miRNAs from the Dlk1-Dio3 locus in human lupus PBMCs (miR-134, miR-382, miR-409, miR-411, miR-493, miR-494, miR-544, and miR-654) [74,76], in circulating blood of patients with class IV lupus nephritis (miR-485-5p, miR-543, miR-410-3p, and miR-369-5p) [123], in the renal biopsy samples of the patients with lupus (miR-134, miR-433, and miR-494) [37,124], and in the lesioned skin biopsy samples of the patients with discoid cutaneous lupus (miR-485-3p) [125]. While miR-654 and miR-134 were upregulated in the urinary exosomes of type IV lupus nephritis patients with cellular crescent [126], miR-654 was found to be decreased in the human lupus PBMCs [127]. Another Dlk1-Dio3 miRNA that was reported to be reduced in the human lupus PBMCs is miR-379 [74]. Recently, Omidi et al. reviewed the lupus-related miRNA profiling studies and arrays in the Gene Expression Omnibus (GEO) database [128]. They discovered nine signature lupus-related miRNAs, of which two (miR-134 and miR-409) are from the Dlk1-Dio3 locus [128].

DNA methylation plays an essential role in genomic imprinting. The dysregulation of Dlk1-Dio3 miRNAs in human pathological conditions has been associated with the methylation change of different DMRs located at the Dlk1-Dio3 region. The overexpression of Dlk1-Dio3 miRNAs in acute promyelocytic leukemia (APL) has been associated with the hypermethylation of conserved binding sites for the CCCTC-binding factor (CTCF, an enhancer blocking protein) at MEG3-DMR [130]. CTCF usually binds to unmethylated DNA to suppress target gene expression. In addition, reduced expression of Dlk1-Dio3 miRNAs, specifically in islet β cells of human patients with type 2 diabetes mellitus (T2DM), has been associated with DNA hypermethylation at MEG3-DMR in the β cells from T2DM donors [22]. Our study found that the upregulation of Dlk1-Dio3 miRNAs in the splenocytes and purified splenic CD4^+^ T cells and CD19^+^ B cells of MRL-*lpr* lupus cells was correlated with DNA hypomethylation in these cells [98]. Demethylation treatment of splenic cells with DNA methylation inhibitor 5-Aza-2′-deoxycytidine significantly increased Dlk1-Dio3 miRNAs expression in the cells from control MRL mice, but not in those from MRL-*lpr* lupus mice. Further, we found that in vitro inhibition of specific Dlk1-Dio3 miRNAs such as miR-154, miR-379, and miR-300 in MRL-*lpr* splenocytes suppressed the production of LPS-induced, lupus-related cytokines such as IFNγ, IL-1β, IL-6, and IL-10 [98]. These data suggest that DNA methylation plays a vital role in regulating genomic imprinting Dlk1-Dio3 miRNAs, which are involved in the regulation of inflammation in autoimmune lupus.

## 8. Potential Mechanism of the Dlk1-Dio3 miRNAs Involved in Autoimmune Disease Pathogenesis

The dysregulation of Dlk1-Dio3 miRNAs has been identified in various types of cancers [99,115]. The Dlk1-Dio3 miRNAs have been implicated in the pathogenesis of cancers via targeting key genes in cancer biology, such as phosphatase and tensin homolog (*PTEN*) [131] and mesenchymal-epithelial transition factor (*c-Met*) [115], and tumorigenesis signaling pathways such as PI3K/AKT [132], Hippo signaling, and p53 signaling [116].

Dlk1-Dio3 miRNAs have been recently implicated in the pathogenesis of autoimmune disease MS and EAE [103,117]. Compared to EAE-resistant rat strains, miRNAs from the Dlk1-Dio3 locus, including miR-127, miR-434, and miR-136, were significantly upregulated in EAE susceptible rat strains after EAE induction. The pathway analysis revealed that these dysregulated miRNAs encoded by the *anti-Rtl1* gene regulates multiple signaling cascades involved in autoimmunity such as signaling pathways regulating T cell proliferation and activation [117]. Furthermore, twenty-six Dlk1-Dio3 miRNAs were identified to be significantly upregulated in the male patients with relapsing-remitting multiple sclerosis (RRMS) compared to those of healthy controls [103]. A network-based enrichment analysis of the target genes of these 26 Dlk1-Dio3 miRNAs was performed. Twenty-four signaling pathways were enriched with target genes of more than six miRNAs. Of these 24 signaling pathways, 17 are highly overlapped and belong to pathways activated through receptor tyrosine kinases. The majority of these pathways regulate PI3K/Akt signaling directly or indirectly. This study suggested that Dlk1-Dio3 miRNAs are involved in male RRMS pathogenesis via regulating PI3K/Akt signaling [103].

An analysis of the predicted miRNA-mediated regulation of 72 lupus susceptibility genes in humans and mice revealed that miRNAs located at the Dlk1-Dio3 locus were able to regulate 48 lupus susceptibility genes such as B cell lymphoma 2 (*BCL2*), *PTEN*, Roquin (*RC3H1*), Bcl-2 like protein 11 (*BIM*), and *TLR7* [133]. Our previous miRNA microarray profiling study identified that selected miRNAs from the Dlk1-Dio3 locus were significantly upregulated in lupus cells, which included miR-127, miR-154, miR-299, miR-300, miR-329, miR-376b, miR-379, miR-382, miR-411, miR-433, and miR-494 [77,98]. Further study is needed to determine the genes and signaling pathways that are targeted by these dysregulated Dlk-Dio3 miRNAs in lupus cells, which is important for understanding the mechanism of Dlk1-Dio3 miRNAs in SLE pathogenesis.

## 9. Conclusions and Perspective

The epigenetic regulation in human SLE etiology has received increased attention in recent years. In this review, we discuss the contribution of DNA methylation and miRNAs to lupus individually, as well as the interplay and cross-regulation between these two epigenetic factors in lupus. In SLE, several miRNAs such as miR-21, miR-148a, and miR-126 have been implicated in the pathogenesis, which affect DNA methylation by targeting DNMTs or signaling components of the DNA methylation signaling pathway. Further, the dysregulation of some miRNAs such as miR-126 [89] and Dlk1-Dio3 miRNAs [98] is correlated with DNA hypomethylation in lupus cells. Further investigation is needed to determine the negative feedback loop between the specific epigenetically regulated miRNA and DNMTs in lupus cells.

A characteristic epigenetic feature of lupus is DNA hypomethylation, particularly in CD4^+^ T cells. The mechanism underlying DNA hypomethylation in lupus could be cell type-specific and signaling pathway-dependent. Both the DNMTs-mediated passive demethylation pathway and the TETs-mediated active demethylation pathway play a role in DNA hypomethylation in lupus. The DNMTs- and TETs-mediated methylation and demethylation of specific gene regulatory regions may rely on crosstalk with specific transcription factors at the gene regulatory sites [134]. It has been reported that during Th differentiation, TET2 was recruited to the Th signature cytokine genes promoter by lineage specific transcription factors such as T-bet, RoRγT, and STAT3, leading to the demethylation and expression of Th specific cytokines such as IFNγ, IL-17, and IL-10 [135]. Further, the transcription factor CTCF has been shown to play an important role in TETs-mediated DNA hydroxymethylation and activation of the suppressor of the cytokine signaling 1 (SOCS1) gene in human lupus CD4^+^ T cells [62]. Moving forward with the current knowledge on the global DNA hypomethylation in lupus, future studies in SLE should examine the crosstalk between specific transcription factors and DNMTs/TETs in specific cell types to determine a cell-specific epigenetic regulation pattern of gene expression at the genome level.

The disruption of genomic imprinting and imprinting gene expression have been implicated in the pathogenesis of human diseases including genetic imprinting disorders [136,137], cancers [138], neurological disorders [139], and autoimmune disorders [117,140,141]. The epigenetic dysregulation of genomic imprinting Dlk1-Dio3 miRNAs in autoimmune diseases such as MS [103,117,141], lupus [98], and T2DM [22] has been reported [98,117]. We have shown that different Dlk1-Dio3 miRNAs were upregulated at varied levels in specific lymphocyte subsets [98]. Further, we noted that there was a differential DNA methylation sensitivity of Dlk1-Dio3 miRNAs in various immune cell subsets [98]. However, whether there is a direct link between Dlk1-Dio3 miRNA expression and the differential DNA methylation at specific regulatory regions of Dlk1-Dio3 domains (such as IG-DMR and Glt2-DMR) remains unknown. Therefore, a high throughput methylation profiling study is necessary to define the differentially methylated sites at specific Dlk1-Dio3 domains such as DMRs and/or CpG enriched regions located at the major miRNA coding regions in the cells of lupus-prone and control mice. This shall provide further evidence supporting the DNA methylation regulation of Dlk1-Dio3 miRNAs in lupus. While the dysregulation of select Dlk1-dio3 miRNAs has been identified in different types of biological samples from human lupus patients and lupus-prone mice (Table 1), the functional significance and the mechanism of the Dlk1-Dio3 miRNA in lupus remains unknown. This need to be addressed in future studies. Altogether, these findings will enhance the current understanding of the epigenetic mechanism in SLE and provide a new perspective for lupus treatment.

## Figures and Tables

**Table 1:** Dysregulated miRNAs from the Dlk1-Dio3 locus in murine and human lupus.

miRNA ID	Expression Change	Sample Source and Type	Method of Detection	Reference
miR-154, miR-127, miR-379, miR-382, miR-433, miR-300, miR-376b, miR-394, miR-299, miR-495, and miR-329	Up	MRL-*lpr* splenocyte	Microarray	[77]
miR-154, miR-127, miR-379, miR-382, miR-300, miR-433, and miR -411	Up	MRL-*lpr* Splenocyte CD4^+^ T CD8^+^ B	RT-qPCR	[98]
miR-127 and miR-379	Up	NZBWF1 splenocyte	RT-qPCR	[77]
miR-127 and miR-379	Up	C3.MRL-Fas^lpr^/J splenocyte	RT-qPCR	[122]
miR-494 and miR-544	Up	Human lupus PBMCs	Taqman low density array	[74]
miR-134, miR-382, miR-409, miR-411, miR-493, miR-494, miR-544, and miR-654	Up	Human lupus PBMCs	Small RNA-seq	[76]
miR-494	Up	Human lupus Naïve B	Taqman array	[129]
miR-485-5p, miR-543, miR-410-3p, and miR-369-5p	Up	Human lupus Type IV nephritis plasma	Small RNA-seq	[123]
miR-654 and miR-134		Human lupus Type IV nephritis with cellular crescent Urinary exosome	Small RNA-seq	[126]
miR-134, miR-433, and miR-494	Up	Human lupus nephritis renal biopsy	Microarray	[124]
miR-485-3p	Up	Human discoid cutaneous lupus skin biopsy	Taqman array	[125]
miR-379	Down	Human lupus PBMCs	Taqman low density array	[74]
miR-654	Down	Human lupus PBMCs	RT-qPCR	[127]

## Data Availability

Not applicable.
